# Hybrid strains of enterotoxigenic/Shiga toxin-producing Escherichia coli, United Kingdom, 2014–2023

**DOI:** 10.1099/jmm.0.001946

**Published:** 2025-01-22

**Authors:** Ella V. Rodwell, David R. Greig, Suzanne Gokool, Israel Olonade, Craig Swift, Yung-Wai Chan, Claire Jenkins

**Affiliations:** 1 Gastrointestinal Infection and Food Safety (One Health) Division, UK Health Security Agency, Colindale, London, UK; 2 NIHR Health Protection Research Unit in Gastrointestinal Infections, University of Liverpool, Liverpool, UK; 3 Gastrointestinal Bacterial Reference Unit, UK Health Security Agency, Colindale, London, UK

**Keywords:** enterotoxigenic *Escherichia coli*, hybrid diarrhoeagenic *E. coli*, outbreak, Shiga toxin-producing *Escherichia coli*, surveillance

## Abstract

**Introduction.** Diarrhoeagenic *Escherichia coli* (DEC) pathotypes are defined by genes located on mobile genetic elements, and more than one definitive pathogenicity gene may be present in the same strain. In August 2022, UK Health Security Agency (UKHSA) surveillance systems detected an outbreak of hybrid Shiga toxin-producing *E. coli*/enterotoxigenic *E. coli* (STEC–ETEC) serotype O101:H33 harbouring both Shiga toxin (*stx*) and heat-stable toxin (*st*).

**Gap statement.** These hybrid strains of DEC are a public health concern, as they are often associated with enhanced pathogenicity. However, little is known about their epidemiology, clinical significance and associated public health burden.

**Aim.** The aim of this study was to describe the microbiology, epidemiology and genomic analysis of this novel hybrid serotype in the context of the STEC–ETEC strains in the UKHSA archive.

**Methodology.** From 2014 to 2023, STEC isolated from faecal specimens testing positive for STEC by PCR were sequenced on the NextSeq 1000 short read platform and a subset were selected for long read nanopore sequencing. Genomes were analysed to determine serotype, *stx* subtype, DEC pathogenicity genes and antimicrobial resistance determinants.

**Results.** There were 162 STEC–ETEC strains isolated between 2014 and 2023, of which 117/162 were human clinical isolates and 45 were of food or animal origin. An average of 16 STEC–ETEC strains were identified each year, exhibiting a range of different *stx* subtypes, the most common profiles being *stx2g,st* (*n*=65, 40%) and *stx2a,st* (*n*=48, 30%). The most common sequence types were ST329 and ST200 (*n*=24 each), and the most frequently detected serotype was O187:H28 (*n*=25). Nine cases of genetically linked STEC–ETEC O101:H33, *stx1a,st* were detected between 8 August and 21 September 2022. Although the temporal and geographical distribution of the cases was characteristic of a foodborne outbreak, the contaminated vehicle was not identified.

**Conclusions.** Phylogenetic analysis and long-read sequencing of the outbreak strain provided insight into the stepwise acquisition of *st* and *stx* and the evolutionary history of STEC–ETEC pathotypes. The integration of epidemiological data and whole-genome sequencing for routine surveillance of gastrointestinal pathogens is key to understanding the emergence of zoonotic hybrid DEC pathotypes and monitoring foodborne threats to public health.

## Introduction

In addition to the extraintestinal pathogenic *Escherichia coli* pathotype, there are five well-established pathotypes of *E. coli* that can cause gastrointestinal symptoms in humans, known as diarrhoeagenic *E. coli* (DEC) [1,2]. The DEC pathotypes are defined by the presence of specific pathogenicity genes, including Shiga toxin-producing *E. coli* (STEC; defined by the presence of one or more Shiga toxin genes, *stx*), enteropathogenic *E. coli* (EPEC; defined by the presence of the *E. coli* attaching and effecting gene, *eae*), enterotoxigenic *E. coli* [ETEC; defined by the presence of heat-labile (*lt*) and/or heat-stable toxin genes (*st*)], enteroaggregative *E. coli* (EAEC; defined by the presence of the aggregative adherence regulator gene, *aggR*) and enteroinvasive *E. coli* (EIEC; defined by the presence of *ipaH*) [1,2]. STEC, ETEC and EPEC are zoonotic gastrointestinal pathogens transmitted to humans by the consumption of contaminated food or water, direct contact with infected animals or their environment and person-to-person spread [3]. EIEC and EAEC are more likely to be spread by person-to-person contact, although both pathogens can be transmitted by the consumption of food and water contaminated by human faeces, for example, by an infected food handler [4,5].

The clinical management and public health actions required are dependent on the severity of the symptoms associated with infection. Symptoms of STEC infection include abdominal cramps, vomiting and severe bloody diarrhoea. In 5–15% of cases, the infection can lead to the development of haemolytic uraemic syndrome (HUS), a severe multisystem syndrome characterized by acute kidney injury that can be fatal, particularly in children and the elderly [1,2]. Symptoms of ETEC are characterized by profuse, watery diarrhoea. Gastrointestinal symptoms of EPEC vary, but the diarrhoea is usually persistent. EIEC infection is characterized by rapid onset (24–48 h after exposure) and watery diarrhoea, sometimes accompanied by blood and mucus (dysentery). Symptoms of EAEC infection are most commonly persistent diarrhoea and abdominal pain [1,2].

The pathogenicity genes that define each pathotype are located on mobile genetic elements, and in certain hybrid DEC pathotypes, more than one definitive pathogenicity gene may be present in the same strain [6,7]. These hybrid strains of DEC are a public health concern, as they are often associated with enhanced pathogenicity [8,9]. For example, the aetiological agent of the outbreak of STEC-HUS in Germany in 2011, which caused 3950 cases, 800 cases of HUS and 53 deaths, was a hybrid DEC STEC/EAEC harbouring *stx* and *aggR* [10]. For this reason, the public health surveillance systems monitor the emergence of hybrid DEC, especially those that have *stx*.

In August 2022, UK Health Security Agency (UKHSA) surveillance systems identified a cluster of cases infected with a rare STEC serotype, STEC O101:H33 belonging to clonal complex (CC) 10. Although the cluster of cases was small (*n*=9), the strain was identified as a hybrid DEC harbouring both *stx* and *st*. The aim of this study was to describe the microbiology, epidemiology and genomic analysis of this novel STEC/ETEC hybrid serotype in the context of hybrid strains of STEC/ETEC in the UKHSA archive.

## Methods

### Microbiology and short-read sequencing

Faecal specimens from hospitalized patients and those with community-acquired gastrointestinal infections testing positive for STEC by PCR in the local hospital setting are referred to the Gastrointestinal Bacteria Reference Unit, UKHSA, for confirmation and culture. Genomic DNA was extracted from DEC isolates and sequenced on Illumina HiSeq 2500 and NextSeq 1000 platforms. Post whole-genome sequencing (WGS), isolates were processed through an in-house pipeline that determines serotype, *stx* subtype, DEC pathogenicity genes (specifically *eae, lt, st, aggR* and *ipaH*) and antimicrobial resistance determinants using GeneFinder (https://github.com/phe-bioinformatics/gene_finder) (Technical Appendix in the Supplementary Material) [11,12]. Single nucleotide polymorphism (SNP) typing using *E. coli* K-12 (U00096.2) as the reference genome was performed, as previously described [13]. All sequences in this study can be found at the Pathogens BioProject (National Center for Biotechnology Information Project No. PRJNA315192).

### Long-read sequencing and data processing

To investigate the location and genomic architecture of *st*-encoding plasmid and the *stx*-encoding prophages, eight isolates of STEC/ETEC O101:H33, including three outbreak isolates, were selected for nanopore sequencing (Table S1 available in the online Supplementary Material), as previously described [14] (Technical Appendix in the Supplementary Material). High-molecular weight (HMW) genomic DNA was extracted using the Fire Monkey HMW DNA extraction kit (Revolugen), and sequencing was performed on a FLO-MIN106 (R9.4.1D) flow cell and a MinION Mk1C (Oxford Nanopore Technologies) for 24 h. Base calling of raw FAST5 data was performed using the Guppy v6.5.7 FAST model. Read trimming, filtering and assembly were performed using Porechop v0.2.4 (https://github.com/rrwick/Porechop), Filtlong v0.2.0 (https://github.com/rrwick/Filtlong) and Flye v2.9, respectively.

*Stx*-encoding prophages were detected and extracted using PhageBoost and Propi v0.0.1, re-annotated using PGAP (build6771) and aligned and compared using Clinker v0.0.27, as previously described [14]. Plasmids were identified in Nanopore-based assemblies as closed circular contigs with a single plasmid replicon. Plasmid replicon detection was performed using PlasmidFinder v.2.135 with the Enterobacteriaceae, minimum identity=90% and minimum coverage=90% parameter set. Annotations from PGAP (build6771) were used with BRIG v0.95 to visualize the IncFIB plasmid in the dataset [14].

### Data deposition

All FASTQ files and assemblies were submitted to the NCBI. Illumina FASTQ and Nanopore FASTQ accessions can be found under BioProject: PRJNA315192 (Table S1).

## Results

### Epidemiology and microbiology of hybrid STEC–ETEC strains in the UKHSA archive

**Fig. 1.**
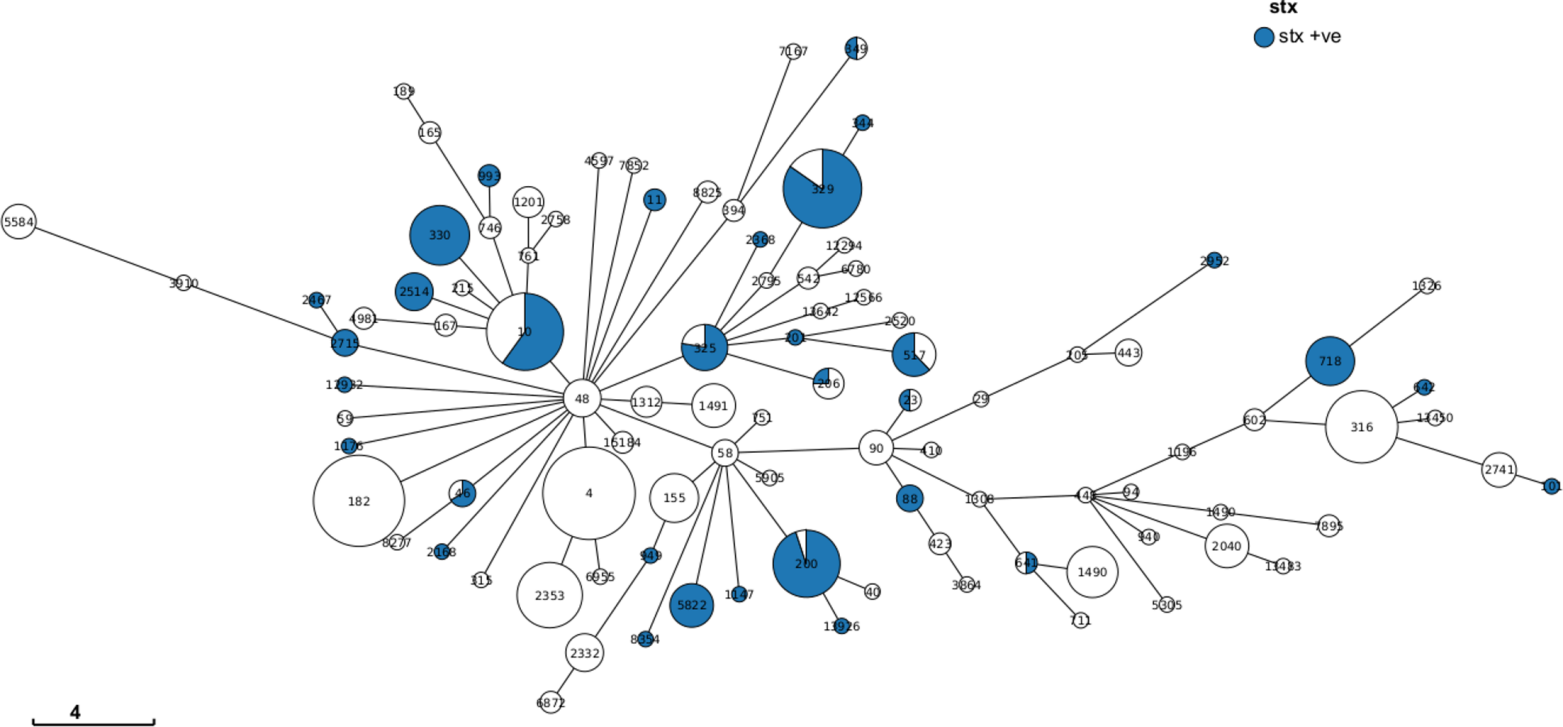
Minimum spanning tree describing the 7-gene Multilocus Sequence Type (MLST) of ETEC samples in the UKHSA archives. Annotated with the proportion of genomes that harboured *stx* (STEC), where MLST profile was available. Labels describe the ST, and colours reflect the presence of any *stx* subtype in line with the legend.

**Fig. 2.**
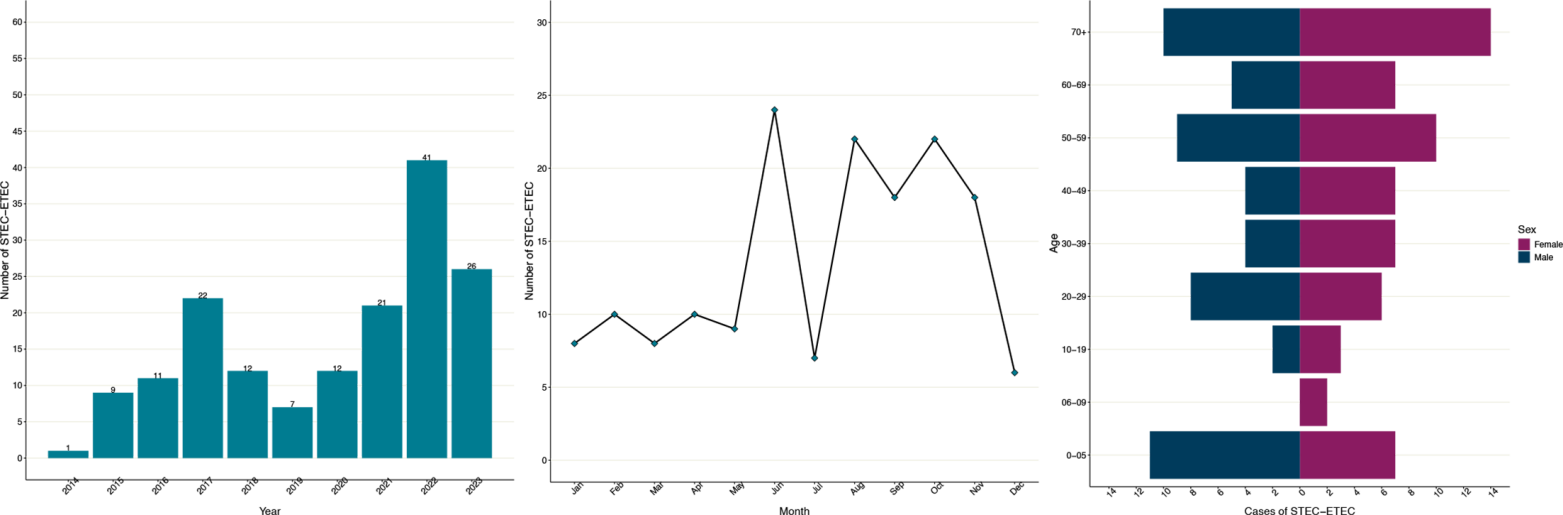
Epidemiology of STEC–ETEC isolates in the UKHSA archives between 2014 and 2023. The three-panelled figure displays the number of notifications of STEC–ETEC (left), the seasonality of STEC–ETEC (middle) and the age–sex distribution of clinical STEC–ETEC (right).

During the time frame of the study, there were 35 265 sequences from isolates of DEC in the UKHSA database. Of these, 534/35 265 (1.5%) were from food and 303/35 265 (0.9%) were from animals. Analyses of the strains of ETEC in the UKHSA archive identified *stx* in 162 isolates (Figs1  2 and Table S1). Between 2014 and 2023, the average number of isolations of hybrid strains of STEC–ETEC each year was 16 (minimum=1; maximum=41), with a seasonal peak in the summer and autumn (Fig. 2). The most common sequence types were ST329 and ST200 (*n*=24 each) and ST330 and ST10 (*n*=16 each). The most frequently detected serotypes were O187:H28 (*n*=25), O136:H12 (*n*=15), O101:H33 (*n*=14), O2:H27 (*n*=16) and O168:H8 (*n*=12) (Table S1). The STEC–ETEC hybrid strains exhibited a wide range of combinations of *stx* types, with the most common pathogenicity profiles being *stx2g, st* (*n*=65, 40%) and *stx2a, st* (*n*=48, 30%) (Table S1).

There were 117/162 clinical isolates from human cases resident across the UK; the majority were female (63/117, 54%) and adults (93/117, 79%) (Fig. 2). There were 45 non-clinical isolates (food, *n*=36; water, *n*=8; animal, *n*=1). Food samples included raw milk (*n*=20), flour (*n*=5), hard cheese made from raw milk (*n*=4), cheese (pasteurization unspecified) (*n*=1), beansprouts (*n*=1) and sprouts (*n*=1) (Table S1).

### Outbreak of STEC–ETEC O101:H33

**Fig. 3.**
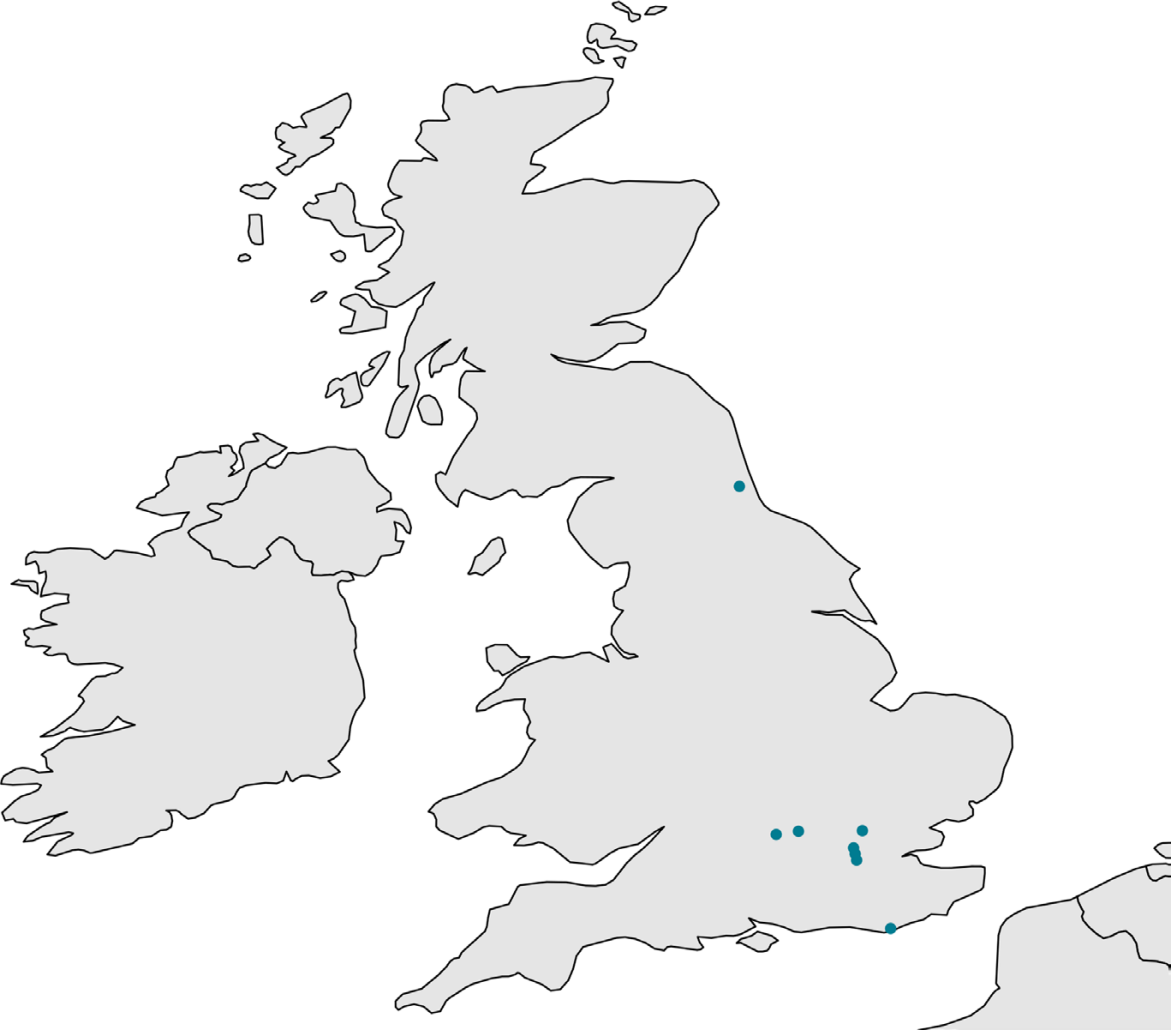
Geographical distribution of STEC–ETEC 101:H33 outbreak cases.

There were nine cases of STEC–ETEC O101:H33, *stx1a,* belonging to a five-SNP single-linkage cluster within CC10, detected between 8 August and 21 September 2022. The nine cases were geographically dispersed across the England (Fig. 3); the majority were female (*n*=8; 89%), and ages ranged from 5 to 67 years with a median of 35 years. Clinical outcome data were available for six of nine cases, all six cases reported diarrhoea and abdominal pain, and three reported bloody stools. All six cases visited the General Practioner; none were hospitalized. In-depth questionnaires administered by telephone interviews captured information on food exposure and travel history. No common food exposures, including eating outside the home, or common travel destinations were identified. Although the temporal and geographical distribution of the cases and lack of common animal and environmental exposures were characteristics of a foodborne outbreak of a nationally distributed product, the contaminated food vehicle was not determined.

### Genomic analysis of *E. coli* O101:H33

**Fig. 4.**
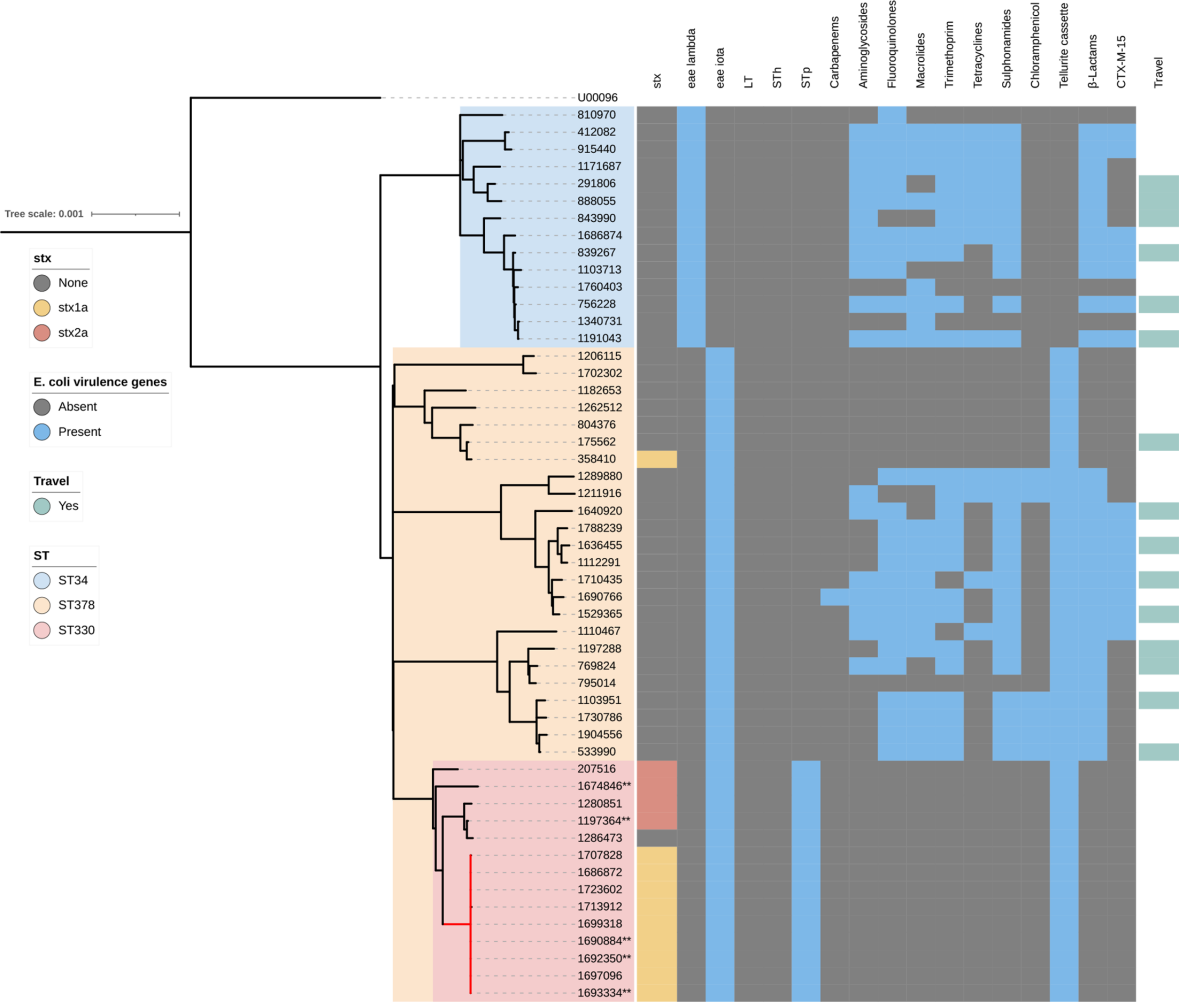
Maximum-likelihood phylogeny of clonal complex 10, serotype O101:H33 (*n*=53) (midpoint rooted) showing genome-derived virulence genes and antimicrobial resistance and reported travel abroad linked to each case. Outbreak isolates (*n*=9) are indicated in a red label, and isolates sequenced using Oxford Nanopore Technology (ONT) have a double asterisk (**). LT Heat Labile toxin; STh Heat Stable (human variant); STp Heath Stable (porcine variant). Colours of the clade indicate sequence type (ST) (blue: ST34; yellow: ST378; pink: ST330).

**Fig. 5.**
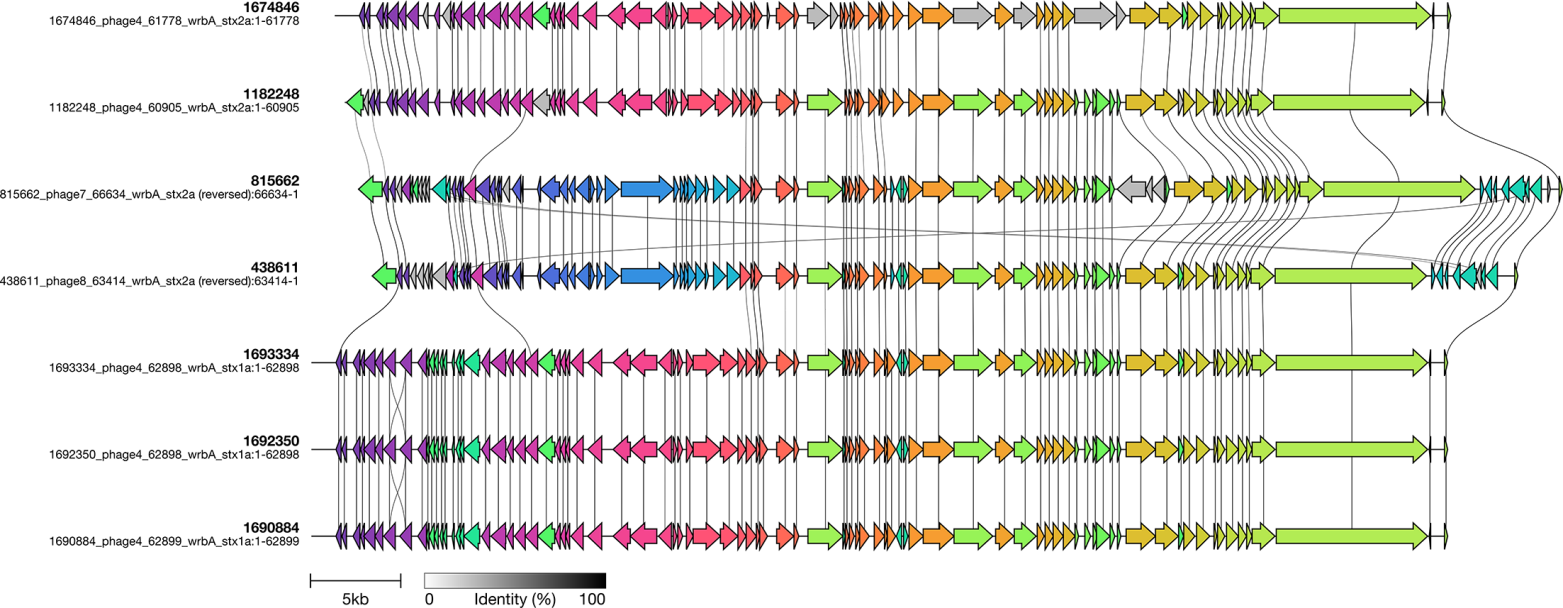
Pairwise alignment of *stx*-encoding prophages. Annotations detail sample ID, prophage number, prophage size (bp), SBI and *stx* subtype.

**Fig. 6.**
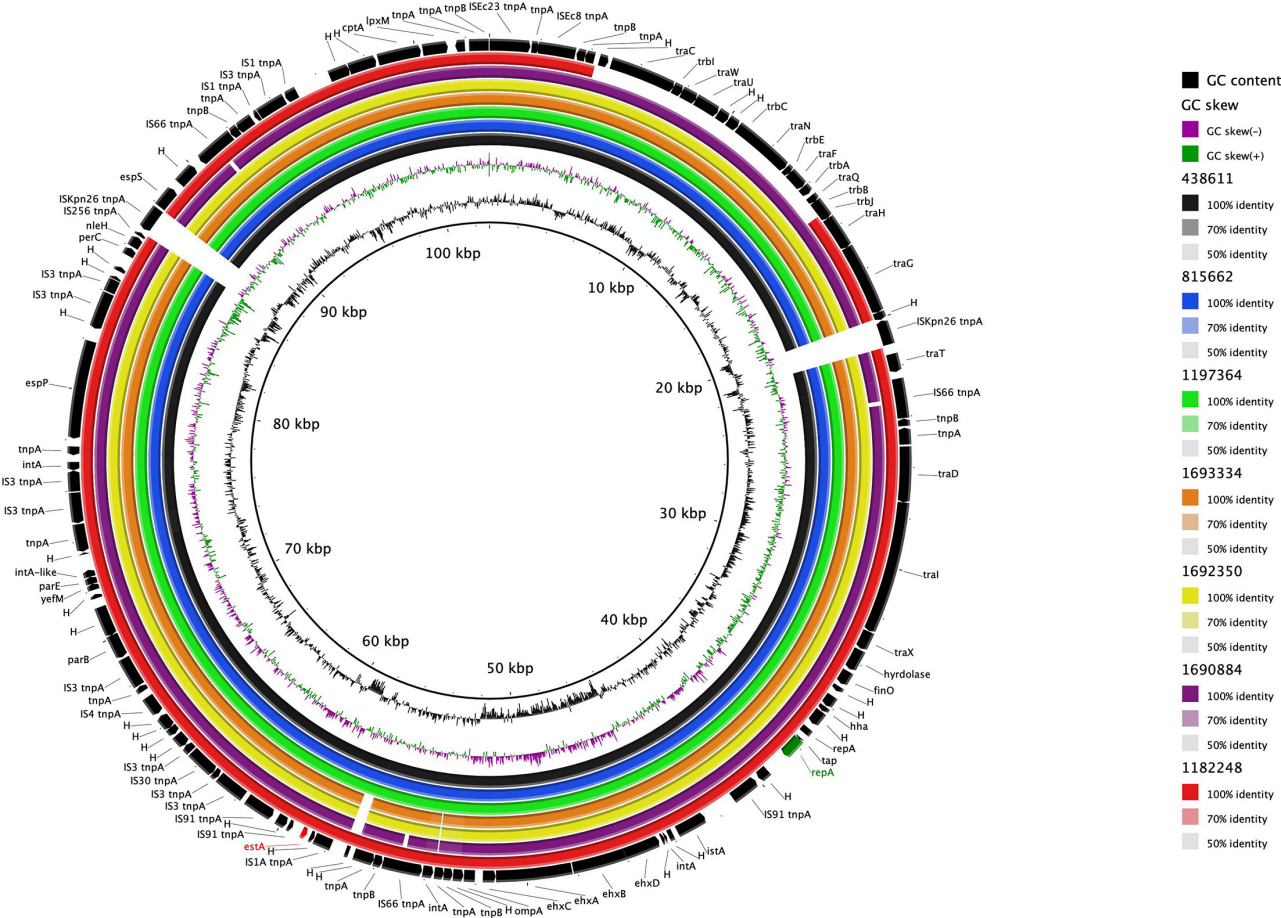
BLAST Ring Image Generator (BRIG) plot comparing IncFII plasmids discovered during this study via nanopore sequencing with *st* highlighted in red. The sequencing identifer of the isolates are listed in the key. Further typing details are available in Table S1. GC content is the percentage of guanine (G) and cytosine (C) bases in the genome.

In addition to the nine STEC/ETEC hybrid outbreak strains, there were another 44 isolates of *E. coli* O101:H33 in the UKHSA archive. All 53 isolates belonged to a clade of three closely related STs within CC10: ST34 (*n*=14), ST330 (*n*=15) and ST378 (*n*=24) (Fig. 4 and Table S1). All isolates in this clade had either one of two variants of *eae* (*eae*-lambda or *eae*-iota); however, only the isolates belonging to ST330 had *st*. Of the 15 isolates that had *stx*, 14/15 belonged to ST330 and 1/24 belonged to ST378 (Fig. 4). The majority of STEC isolates had *stx1a* (*stx1a*, *n*=10/15; *stx2a*=5/15). Phylogenetic analysis showed that the nine STEC/ETEC hybrid outbreak isolates fell within ST330 and had *eae*-iota. None of the ST34 isolates had *stx*. Nanopore sequencing of eight STEC–ETEC isolates belonging to ST330, including three outbreak isolates, revealed that the *stx*-encoding prophages exhibited sequence variation, although they were all inserted at the same site, *wrbA*, a well-established site of bacteriophage insertion in STEC (SBI) (Fig. 5) [15]. We also observed that *st* was encoded on a 97.3–97.5 kb IncFII plasmid (Fig. 6). Like the *st* in the STEC/ETEC isolate belonging to ST330 described by Nyholm *et al*. [16] (IH53473), we found that the *st* in the STEC/ETEC isolate belonging to ST330 in our study had a frameshift mutation resulting in a premature stop codon.

Travel history was recorded for 19/53 (36%) cases, of which 15/53 (28%) cases reported travelling outside the UK. The travel-related cases belonged to either ST34 (Columbia *n*=2, Egypt *n*=2, Morocco *n*=1, Turkey *n*=1) or ST378 (Pakistan *n*=4, India *n*=2, Mexico *n*=1, Nepal=1, destination not recorded=1). All the travel-related isolates had *eae* but not *stx* or *lt*/*st* (Fig. 4). Of the isolates belonging to the travel-associated STs (ST34 and ST378), 27/38 (71%) were multidrug-resistant, exhibiting resistance to three or more classes of antimicrobial. Fifteen of the 38 isolates in the travel-associated STs had the extended-spectrum beta-lactamase gene, *bla*_CTX-M-15_ (Fig. 4).

## Discussion

Notifications of hybrid strains of STEC–ETEC in the UK remain relatively low compared with other DEC pathotypes [17]. Despite the improvements in molecular diagnostics for DEC over the last decade, the analyses revealed a fluctuating trend in case numbers of STEC/ETEC [18]. In line with other gastrointestinal pathogens, there was a decrease in case numbers during the COVID-19 pandemic, followed by a steep increase in 2022 once lockdown restrictions were relaxed [19]. Strains of STEC–ETEC exhibit both heat-stable toxin and a wide variety of Shiga toxin subtypes, including *stx2a*. Cases infected with STEC harbouring *stx2a* are more likely to report symptoms at the severe end of the spectrum and be associated with progression to HUS [8]. However, clinical outcome data were unavailable for most of the cases, and we were unable to assess the clinical burden associated with this hybrid pathotype.

Although this is the first report of hybrid strains of STEC–ETEC in the UK, previous studies have described strains of STEC–ETEC isolated from food, ruminants and swine [20,29]. Hybrid strains of STEC–ETEC have been described elsewhere in Europe [20,23], Africa [24,25] and Asia [26,29]. Here, we present further evidence that hybrid strains of STEC–ETEC are zoonotic and cause foodborne gastrointestinal infections. There were 45 isolates of food or animal origin in the UKHSA archive, and although we were unable to identify the vehicle of infection of the STEC–ETEC O101:H33 outbreak described in this study, the widespread geographical location of the cases is suggestive of a foodborne source.

Analysis of the deeper phylogenetic context of the outbreak cluster identified three closely related sequence types. Two of the STs comprised a high proportion of cases of EPEC reporting travel outside the UK in the days prior to the onset of symptoms and were characterized by multidrug resistance, including *bla*_CTX-M-15_. Previous studies have associated multidrug resistant (MDR) DEC with travellers’ diarrhoea [17,30]. The phylogenetic analysis described here enabled us to explore the evolutionary dynamics of emerging pathogenic variants of hybrid DEC and speculate on the stepwise acquisition of virulence genes. However, from our analysis, we were unable to determine whether the IncFII plasmid or the *stx*-encoding phage was acquired first, or if they were acquired at the same time. We also considered the possibility that the *stx*-encoding phage may have been acquired on the IncFII plasmid and subsequently incorporated into the chromosome [31].

While notifications of hybrid strains of STEC–ETEC in the UK remain relatively low compared with other DEC pathotypes, this study provided evidence that foodborne outbreaks can occur. Phylogenetic analysis and long-read sequencing of the outbreak strain revealed that ancestral strains of EPEC subsequently acquired both bacteriophage-encoded *stx* and plasmid-encoded *st*, thus providing insight into the stepwise acquisition of *st* and *stx* and the evolutionary history of STEC–ETEC pathotypes. The integration of epidemiological data and WGS for routine surveillance of gastrointestinal pathogens is key to understanding the emergence of zoonotic hybrid DEC pathotypes and monitoring foodborne threats to public health.

## supplementary material

10.1099/jmm.0.001946 Uncited Table S1.

## Abbreviations

CC: clonal complex
DEC: diarrhoeagenic *Escherichia coli*
EAEC: enteroaggregative *Escherichia coli*
EIEC: enteroinvasive *Escherichia coli*
EPEC: enteropathogenic *Escherichia coli*
ETEC: enterotoxigenic *Escherichia coli*
HUS: haemolytic uraemic syndrome
MLST: multilocus sequence type
SNP: single nucleotide polymorphism
STEC: Shiga toxin-producing *Escherichia coli*
UKHSA: UK Health Security Agency
WGS: whole-genome sequencing

## References

[1] Croxen MA, Law RJ, Scholz R, Keeney KM, Wlodarska M (2013). Recent advances in understanding enteric pathogenic *Escherichia coli*. Clin Microbiol Rev.

[2] Nataro JP, Kaper JB (1998). Diarrheagenic *Escherichia coli*. Clin Microbiol Rev.

[3] Kolenda R, Burdukiewicz M, Schierack P (2015). A systematic review and meta-analysis of the epidemiology of pathogenic *Escherichia coli* of calves and the role of calves as reservoirs for human pathogenic *E. coli*. Front Cell Infect Microbiol.

[4] Jenkins C (2018). Enteroaggregative *Escherichia coli*. Curr Top Microbiol Immunol.

[5] Newitt S, MacGregor V, Robbins V, Bayliss L, Chattaway MA (2016). Two Linked *Enteroinvasive Escherichia coli* outbreaks, Nottingham, UK, June 2014. Emerg Infect Dis.

[6] Tozzoli R, Grande L, Michelacci V, Ranieri P, Maugliani A (2014). Shiga toxin-converting phages and the emergence of new pathogenic *Escherichia coli*: a world in motion. Front Cell Infect Microbiol.

[7] Hazen TH, Michalski J, Luo Q, Shetty AC, Daugherty SC (2017). Comparative genomics and transcriptomics of *Escherichia coli* isolates carrying virulence factors of both enteropathogenic and enterotoxigenic *E. coli*. Sci Rep.

[8] Njamkepo E, Fawal N, Tran-Dien A, Hawkey J, Strockbine N (2016). Erratum: Global phylogeography and evolutionary history of *Shigella dysenteriae* type 1. Nat Microbiol.

[9] Koutsoumanis K, Allende A, Alvarez‐Ordóñez A, Bover‐Cid S, Chemaly M (2020). Pathogenicity assessment of Shiga toxin‐producing *Escherichia coli* (STEC) and the public health risk posed by contamination of food with STEC. EFS2.

[10] Kampmeier S, Berger M, Mellmann A, Karch H, Berger P (2018). The 2011 German *Enterohemorrhagic Escherichia coli* O104:H4 outbreak-the danger is still out there. Curr Top Microbiol Immunol.

[11] Chattaway MA, Dallman TJ, Gentle A, Wright MJ, Long SE (2016). Whole genome sequencing for public health surveillance of Shiga toxin-producing *Escherichia coli* other than serogroup O157. Front Microbiol.

[12] Dallman TJ, Byrne L, Ashton PM, Cowley LA, Perry NT (2015). Whole-genome sequencing for national surveillance of Shiga toxin-producing *Escherichia coli* O157. Clin Infect Dis.

[13] Dallman T, Ashton P, Schafer U, Jironkin A, Painset A (2018). SnapperDB: a database solution for routine sequencing analysis of bacterial isolates. Bioinformatics.

[14] Greig DR, Quinn OI, Rodwell EV, Olonade I, Swift C (2024). Genomic analysis of an outbreak of Shiga toxin-producing *Escherichia coli* O183:H18 in the United Kingdom, 2023. Microb Genom.

[15] Yara DA, Greig DR, Gally DL, Dallman TJ, Jenkins C (2020). Comparison of shiga toxin-encoding bacteriophages in highly pathogenic strains of shiga toxin-producing escherichia coli O157:H7 in the UK. Microb Genom.

[16] Nyholm O, Halkilahti J, Wiklund G, Okeke U, Paulin L (2015). Comparative genomics and characterization of hybrid Shigatoxigenic and enterotoxigenic *Escherichia coli* (STEC/ETEC) strains. PLoS One.

[17] Boxall MD, Day MR, Greig DR, Jenkins C (2020). Antimicrobial resistance profiles of diarrhoeagenic *Escherichia coli* isolated from travellers returning to the UK, 2015-2017. J Med Microbiol.

[18] Vishram B, Jenkins C, Greig DR, Godbole G, Carroll K (2021). The emerging importance of Shiga toxin-producing *Escherichia coli* other than serogroup O157 in England. J Med Microbiol.

[19] Love NK, Douglas A, Gharbia S, Hughes H, Morbey R (2023). Understanding the impact of the COVID-19 pandemic response on GI infection surveillance trends in England, January 2020-April 2022. Epidemiol Infect.

[20] Lauzi S, Luzzago C, Chiani P, Michelacci V, Knijn A (2022). Free-ranging red deer (Cervus elaphus) as carriers of potentially zoonotic Shiga toxin-producing *Escherichia coli*. Transbound Emerg Dis.

[21] Nüesch-Inderbinen M, Barmettler K, Stevens MJA, Cernela N (2024). Shiga toxin-producing *Escherichia coli* isolated from hunted wild boar (Sus scrofa) in Switzerland. Schweiz Arch Tierheilkd.

[22] Bai X, Zhang J, Ambikan A, Jernberg C, Ehricht R (2019). Molecular characterization and comparative genomics of clinical hybrid Shiga toxin-producing and enterotoxigenic *Escherichia coli* (STEC/ETEC) strains in Sweden. Sci Rep.

[23] Nyholm O, Heinikainen S, Pelkonen S, Hallanvuo S, Haukka K (2015). Hybrids of Shigatoxigenic and enterotoxigenic *Escherichia coli* (STEC/ETEC) among human and animal isolates in Finland. Zoonoses Public Health.

[24] Ji X, Liang B, Sun Y, Zhu L, Zhou B (2020). An extended-spectrum beta-lactamase-producing hybrid Shiga-toxigenic and enterotoxigenic *Escherichia coli* strain isolated from a piglet with diarrheal disease in northeast China. Foodborne Pathog Dis.

[25] Johura FT, Parveen R, Islam A, Sadique A, Rahim MN (2016). Occurrence of hybrid *Escherichia coli* strains carrying Shiga toxin and heat-stable toxin in livestock of Bangladesh. Front Public Health.

[26] Aref N-EM, Abdel-Raheem A-RA, Kamaly HF, Hussien SZ (2018). Clinical and sero-molecular characterization of *Escherichia coli* with an emphasis on hybrid strain in healthy and diarrheic neonatal calves in Egypt. Open Vet J.

[27] Martikainen O, Kagambèga A, Bonkoungou IJ, Barro N, Siitonen A (2012). Characterization of Shigatoxigenic *Escherichia coli* strains from Burkina Faso. Foodborne Pathog Dis.

[28] Yang X, Liu Q, Bai X, Hu B, Jiang D (2022). High prevalence and persistence of *Escherichia coli* strains producing Shiga toxin subtype 2k in goat herds. Microbiol Spectr.

[29] Yang X, Wu Y, Liu Q, Sun H, Luo M (2021). Genomic characteristics of Stx2e-producing *Escherichia coli* strains derived from humans, animals, and meats. Pathogens.

[30] Dallman TJ, Neuert S, Fernandez Turienzo C, Berin M, Richardson E (2023). Prevalence and persistence of antibiotic resistance determinants in the gut of travelers returning to the United Kingdom is associated with colonization by pathogenic *Escherichia coli*. Microbiol Spectr.

[31] Den Ouden A, Greig DR, Rodwell EV, Tripodo F, Olonade I (2023). *Escherichia coli* encoding Shiga toxin subtype Stx2f causing human infections in England, 2015-2022. J Med Microbiol.

